# Green Synthesis of TiO_2_ Nanoparticles Using Natural Marine Extracts for Antifouling Activity

**DOI:** 10.3390/md21020062

**Published:** 2023-01-19

**Authors:** Walied M. Alarif, Yasser A. Shaban, Mohammed I. Orif, Mohamed A. Ghandourah, Adnan J. Turki, Hajer S. Alorfi, Hermine R. Z. Tadros

**Affiliations:** 1 Department of Marine Chemistry, Faculty of Marine Sciences, King Abdulaziz University, Jeddah 21589, Saudi Arabia; 2 Department of Chemistry, Faculty of Science, King Abdulaziz University, Jeddah 21589, Saudi Arabia; 3 National Institute of Oceanography and Fisheries, Kayet Bay, Alexandria 21556, Egypt

**Keywords:** green synthesis, antifouling formulations, TiO_2_ nanoparticles, marine natural extracts

## Abstract

Titanium dioxide (TiO_2_) nanoparticles were synthesized via a novel eco-friendly green chemistry approach using marine natural extracts of two red algae (*Bostrychia tenella* and *Laurencia obtusa*)*,* a green alga (*Halimeda tuna*), and a brown alga (*Sargassum filipendula*) along with a marine sponge sample identified as *Carteriospongia foliascens*. X-ray diffraction (XRD), scanning electron microscope (SEM), UV–Vis, X-ray spectroscopy (EDS), X-ray photoelectron spectroscopy (XPS), and Fourier-transform infrared spectroscopy (FTIR) were employed to characterize the crystal structure, surface morphology, and optical properties of the synthesized nanoparticles. Each of the as-synthesized marine extract based TiO_2_ nanoparticles was individually incorporated as an antifouling agent to form a newly fabricated marine paint formulation. The newly prepared formulations were applied on unprimed steel panels. A comparative study with a commercial antifouling paint (Sipes Transocean Coatings Optima) was carried out. After 108 days of the coated steel panels’ immersion in the Eastern Harbour seawater of Alexandria-Egypt, the prepared paints using *B. tenella* and *C. foliascens* extracts demonstrated an excellent antifouling performance toward fouling organisms by inhibiting their settlement and controlling their adhesion onto the immersed panels. In contrast, heavy fouling with barnacles was observed on the surface of the coated panel with the commercial paint. The physicochemical parameters of the seawater surrounding the immersed coated panels were estimated to investigate the influence of the fabricated paint formulations. Interestingly, no effects of the immersed coated panels on the physicochemical characteristics of the surrounding seawater were observed. Based on the obtained results and a comparison with commercially available antifouling products, the marine extract based TiO_2_ nanoparticle preparations of *B. tenella* and *C. foliascens* are promising candidates for eco-friendly antifouling agents. Based on the obtained results and a comparison with commercially available antifouling products, the marine extract based TiO_2_ nanoparticle preparations of *B. tenella* and *C. foliascens* are promising candidates for eco-friendly antifouling agents, which could be attributed to the small crystallite sizes of 22.86 and 8.3 nm, respectively, in addition to the incorporation of carbon in the crystal structure of the nanoparticles.

## 1. Introduction

Biological fouling is the undesirable accumulation of various organisms, such as aquatic animals, microbes, plants, and diatoms on submerged artificial surfaces [1]. For many decades, combating biological fouling has been an eternal pursuit. Traditionally, extremely harmful substances, such as tributyltin, arsenic, and mercury were used as antifoulants but because of the risks posed, they have been banned globally [2,3].

Worldwide, more than 80,000 tons of marine antifouling paints, most of which are copper based, are used annually to protect vessels. Unfortunately, these materials still pose a threat to various marine organisms. Furthermore, a serious eco-toxicological concern has been created as a result of the utilization of hazardous chemicals and their release into the environment [4]. Therefore, to completely eliminate this threat, the development of efficient, viable, and environmentally safe coatings that are able to conquer the drawbacks and avoid adverse effects of the current traditional methods is imperative [5].

Red algae, especially many species of the genus *Laurencia* (order Ceramiales, family Rhodomelaceae), are proven to be rich sources of halogenated secondary metabolites. Many of these metabolites have been found to possess a variety of biological activities such as antifeedant (diterpenes), antihelmintic (β-chamigrane-type sesquiterpenes), antimalarial (brominated sesquiterpenes), antifouling (sesquiterpenes), antimicrobial (allolaurinterol) and cytotoxic activities [6]. *L. obtusa* was collected from Cesme coasts (Turkey) was screened by using in vitro methods. The antimicrobial potential of the L. obtusa essential oil was found considerably strong. It presented an inhibitory effect on two bacteria (methicillin-oxacillin resistant *Staphylococcus aureus* ATCC 43300, hemorrhagic *Escherichia coli* (O157: H7) RSSK 232)) and one yeast strain (*Candida albicans* ATCC 10239) [6]. afael et al. (2010) studied the biological properties of specimens from the red alga *B. tenella* J. Agardh (Rhodomelaceae, Ceramiales) extracted by organic solvents. The *n*-hexane (BT-H) and dichloromethane (BT-D) fractions showed antiprotozoal potential and presented high activity in an antifungal assay [7].

The seaweed *H. tuna* was examined for antibacterial and antifungal activity in vitro. The activity study was against 10 bacterial strains and nine fungal strains. The methanolic extract exhibited a broad spectrum of antimicrobial activity. The results confirmed the use of seaweed extracts as a source of an antimicrobial compound [8].

Three selected brown marine algae (*S. denticulatum*, *S. filipendula*, and *Padina gymnospora*) were collected from Al-Shuqaiq province’s Red Sea coast in Saudi Arabia. Their antibacterial activity was evaluated for their potential antibacterial bioactivity against 11 isolates of bacteria isolated from wastewater. The first two algal diethyl ether extracts were the most effective targeted marine algae against wastewater-isolated bacteria [9].

The order Dictyoceratida (Phylum Porifera, Class Demospongiae, Order Dictyoceratida) has contributed over 20% of new secondary metabolites which were previously derived from all sponges, making it the highest producer among all the sponge orders in their biological activities. Their compounds were reported to exhibit different biological activities including cytotoxic, antimicrobial, antiparasitic, anti-*H. pylori*, antiviral, antioxidant, antiallergic, anti-inflammatory, inhibition of atherosclerosis and other activities [10].

In recent decades, scientists have utilized nanomaterials to detect and reduce biofouling activity. Kordas (2019) developed and incorporated CuO, ZnO, and CeMo nanoparticles into commercial paints to examine their properties [11]. His work supported the elucidation that the novel nanomaterial, if incorporated in commercial paints, may achieve a main milestone in the novel descent of marine paints. Scandura and his coworkers (2017) reported a methyl-modified silica xerogel coating functionalized with encapsulated Bi_2_WO_6_. The coating effectively inhibited the accumulation of biomass on glass immersed in seawater [12].

Titanium oxide (TiO_2_) has excellent photocatalytic performance and chemical stability; on the other hand, it is low cost and biologically safe [13]. For these reasons, TiO_2_ has received special attention for its purification applications and as an antimicrobial agent [14,15,16,17]. Conventionally, TiO_2_ nanoparticles were commonly synthesized by various physical and chemical techniques such as solvothermal, reduction, non-sputtering, electrochemical, and sol-gel [18,19,20]. However, these technologies are associated with some limitations and drawbacks, such as high cost, toxicity, and requirements of high energy and pressure [21]. 

On the other hand, previous studies revealed that extracts of algae, corals, sponges, seaweed, and land plants all have active antifouling activity [22]. For example, coated surfaces with dead biomass of *Sepia officinalis* shell incorporated into the paint formulation showed excellent steel surface protection for 200 days of immersion in seawater [23]. The extracts of the Red Sea cucumber species *Holothuria, H. atra*, and *H. nobilis* applied on PVC plates showed good antifouling activity in seawater [24]. Soft coral-derived terpenoids provided the prime chemical protection against naturally occurring predators [25].

Green synthesis has been considered an emerging approach of synthesizing nanoparticles (NPs), due to its simplicity, eco-friendliness, biocompatibility, and economic viability [26]. The synthesis of nanoparticles using plant phytochemicals having antioxidant, or reducing characteristics, can be applied as an effective strategy as it lessens the chances of associated contamination by hazardous chemicals, and helps to cope with environmental pollution.

Therefore, our study aimed at developing environmentally benign, naturally based titanium oxide nanoparticles as antifouling agents for marine vessels and submerged infrastructure. TiO_2_ nanoparticles were synthesized via a novel eco-friendly green chemistry approach using marine natural extracts of *Bostrychia tenella*, *Laurencia obtusa, Halimeda Tuna*, *Sargassum filipendula,* and *Carteriospongia foliascens* (Figure S1) which are denoted as BTiO_2_, LTiO_2_, HTiO_2_, STiO_2_, and CTiO_2_, respectively.

The prepared nanoparticles were applied as antifouling agents in newly prepared marine antifouling paint formulations. The fabricated antifouling paint formulations were applied on steel plates through two successive coatings. After complete drying, the panels were immersed in the Eastern Harbour seawater of Alexandria, Egypt and were followed up by photographic and visual inspections. The characteristics of seawater surrounding the immersed panels were studied at the same time of immersion, as well as at different time intervals to investigate the influence of the fabricated paint formulations.

## 2. Results

### 2.1. CTiO_2_ and BTiO_2_ Characterization

#### 2.1.1. Crystal Phase and Particle Size Analysis

**Figure 1:**
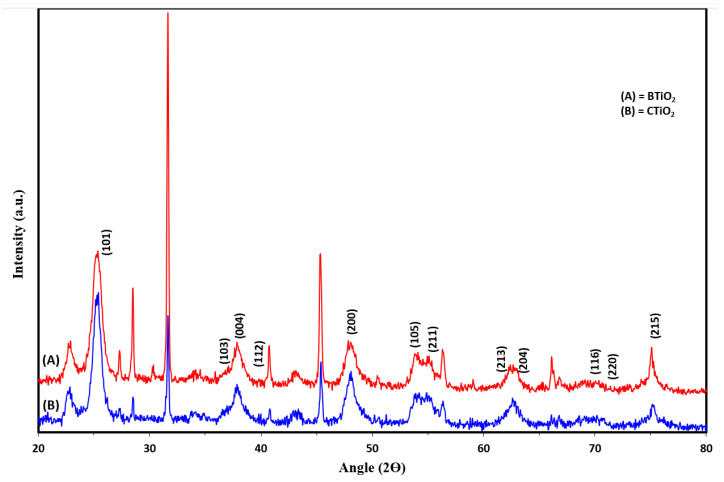
XRD patterns for: (**A**) BTiO_2_ and (**B**) CTiO_2_.

XRD analysis was carried out to determine the crystal phase and the crystallite size of CTiO_2_ and BTiO_2_ nanoparticles. As can be seen from Figure 1, a typical pattern of anatase was indicated for the as-synthesized catalysts by the characteristic diffraction peaks indexed as (101), (103), (004), (112), (200), (105), (211), (213), (204), (116), (220), and (215) for BTiO_2_ and CTiO_2_.

Based on the XRD results, the average crystallite sizes of the catalysts were estimated by using Debye–Scherrer’s equation:

D = k λ/(β cosθ) (1)

where D is the average crystallite size in nm, λ is (0.15418 nm) is the wavelength of X-ray radiation, k is the Scherrer’s constant (k = 0.9), θ is the diffraction angle, and β is a full width at half the maximum (FWHM) of the diffraction line observed. The average crystallite sizes were found to be about 8.3 nm for CTiO_2,_ and 22.86 nm for BTiO_2_.

The smaller particle sizes of BTiO_2_ and CTiO_2_ nanoparticles, estimated from XRD analysis, can be attributed to the incorporation of carbon atoms in both catalysts, which suppressed their crystal growth [27], as a result of the replacement of Ti or/and O in the TiO_2_ lattice by carbon dopant atoms [28] and the segregation of the doped carbon atoms near the grain boundaries, leading to the inhibition of the grain growth of TiO_2_ particles by providing a barrier between them and restricting their direct contact [29].

Physical properties, such as particle size and crystal form of TiO_2_ nanoparticles, can significantly affect their antifouling activity. It has been reported that the algicidal effect of TiO_2_ particles increases with decreasing particle size [30]. Additionally, the biocidal activity of anatase nanoparticles was much higher than that of rutile nanoparticles. Furthermore, the charge carrier mobility and separation of anatase are much better than those of rutile [30]. Accordingly, the as-synthesized BTiO_2_ and CTiO_2_ nanoparticles of the anatase phase with smaller particle sizes may exhibit enhanced antifouling performance.

#### 2.1.2. Surface Morphology

**Figure 2:**
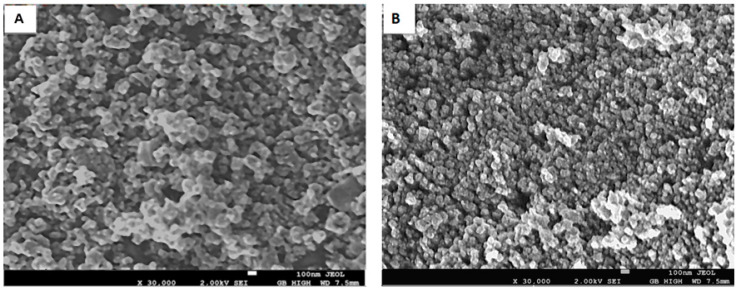
SEM images for: (**A**) BTiO_2_ and (**B**) CTiO_2_.

The SEM images were used to examine the surface morphology of the photocatalysts. The SEM images of CTiO_2_ and BTiO_2_ nanoparticles are shown in Figure 2A. As is clearly evident, the monodispersed particle size distribution for the nanoparticles of both samples were uniform with smaller crystals for C-TiO_2_ (Figure 2B), which is in close agreement with the average crystallite sizes estimated from the XRD patterns.

#### 2.1.3. EDS Analysis

**Figure 3:**
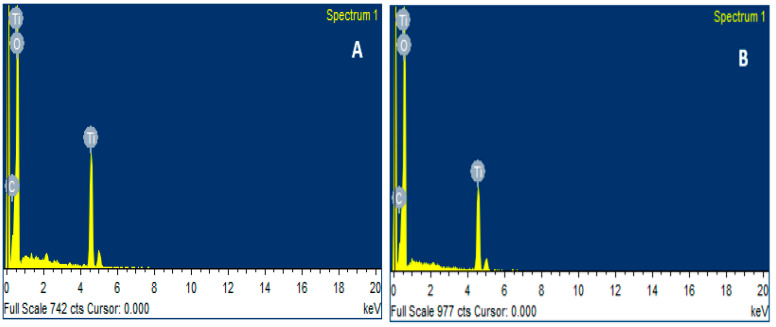
EDS analysis for: (**A**) BTiO_2_ and (**B**) CTiO_2_.

The EDS spectra of the two catalysts are displayed in Figure 3. Well defined peaks for Ti, O, and C elements are shown for both CTiO_2_ and BTiO_2_. The elemental composition of the catalysts determined through the EDS analysis is shown in Table 1. The presence of carbon 13.60 atomic% for BTiO_2_ and 16.91 atomic% for CTiO_2_ nanoparticles reveal the incorporation of carbon into the lattice of both catalysts during the synthesis process via the addition of natural marine extract containing organic compounds. To incorporate C atoms into the TiO_2_ lattice, three possible theatrical scenarios were proposed by Di Valentin and his coworkers [31]. Firstly, the substitution of a lattice oxygen with a carbon; secondly, the replacement of Ti atoms by C atoms. Lastly, stabilization of carbon at an interstitial position.

#### 2.1.4. XPS Analysis

X-ray photoelectron spectroscopy (XPS) was applied to determine the chemical species at the surface of CTiO_2_ and CTiO_2_ nanoparticles.

##### XPS Analysis for CTiO_2_

**Figure 4:**
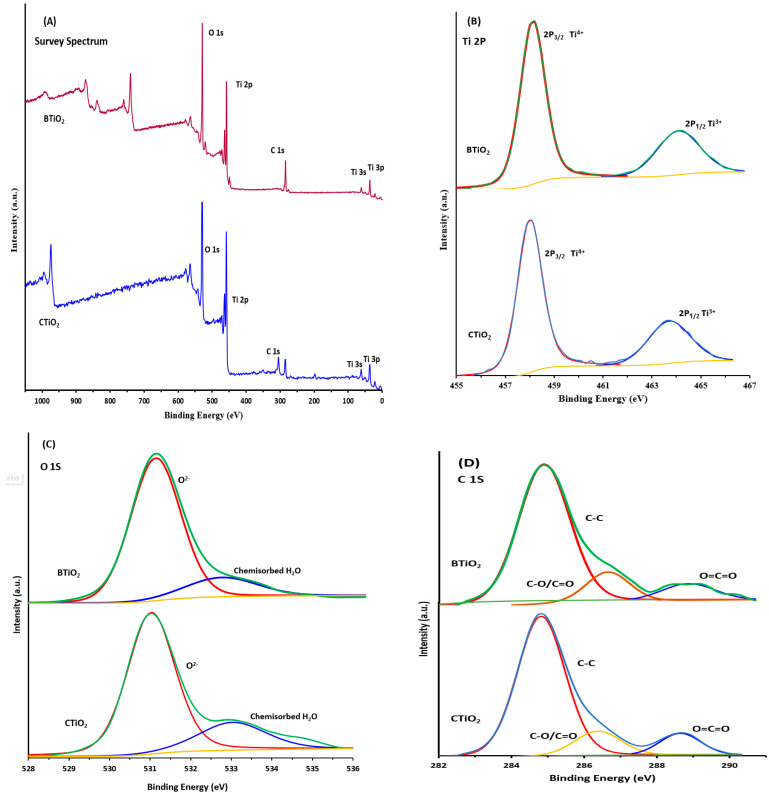
X-ray photoelectron spectroscopy (XPS) for (**A**) survey, (**B**) Ti 2p, (**C**) O 1S, and (**D**) C 1S of BTiO_2_ and CTiO_2_.

The presence of Ti, O, and C in CTiO_2_ has been revealed by the XPS survey spectrum (Figure 4a). The Ti 2p spectrum in Figure 4b shows two well-resolved spin peaks of Ti 2p_3/2_ and Ti 2p_1/2_ at 458.01and 463.7 eV, respectively. The binding energy difference between Ti 2p_3/2_ and Ti 2p_1/2_ is 5.69 eV is within the standard reference value of TiO_2_. The binding energies, as well as spin-orbit divergence, suggest the existence of Ti as Ti^4+^ [32,33]. Figure 4c shows the O 1s XPS spectrum which consists of two peaks; the first binding energy peak at 531.03 eV is ascribed to lattice oxygen. The other binding energy peaks at 533.03 eV and is assigned to surface-adsorbed hydroxyl groups of the chemisorbed H_2_O or the free hydroxyl group (O-H) on the surface [34,35,36,37]. The C 1s XPS spectrum, shown in Figure 4d, reveals three peaks at 284.91, 286.51, and 288.71 eV, which correspond to the carbon of C-C, C-O/C=O, and O-C=O, respectively [38,39]. The XPS results indicate that the atomic composition of CTiO_2_ was found to be 22.9% Ti 2p, 53.4% O 1s, and 23.7% C 1s (Table 1).

##### XPS Analysis for BTiO_2_

The XPS survey spectrum of BTiO_2_ shows the presence of Ti, O, and C (Figure 4A). The Ti 2p spectrum with two spin-orbit components of Ti2p-2p_3/2_ and Ti-2p_1/2_ located at binding energies of 458.00 eV and 463.71 eV, respectively, is demonstrated in Figure 4B. The existence of Ti in the oxidation state of Ti^4+^ is indicated by the 5.71 eV energy separation between Ti2p-2p_3/2_ and Ti-2p_1/2_ [32,33]. Figure 4C displays three peaks for the O 1s. One intense peak at 531.01 eV is attributed to the bulk lattice oxygen [36,37] bound to Ti (Ti-O, Ti-O-Ti). One less intense peak with binding energy at 532.58 eV is assigned to the chemisorbed H_2_O or the free OH on the surface [34,35,36,37]. Three peaks centred at 284.77, 286.44, and 288.57 eV are included in the C 1s XPS spectrum (Figure 4D), suggesting the existence of carbon as C-C, C-O/C=O, and interstitial carbon (O-C=O) in the TiO_2_ lattice, respectively [38,39]. The XPS results indicate that the atomic composition of Ti2p, O1s, and C1s for BTiO_2_ determined by XPS analysis was found to be 19.5% Ti2p, 66.3% O1s, and 14.2% C 1s, respectively, (Table 1).

The atomic% of carbon obtained by EDS (14.2% for BTiO_2_ and 23.7 for CTiO_2_) is quite different than that obtained by XPS (13.60% for BTiO_2_ and 16.91% for CTiO_2_) can be explained based on the differences between the natures of the incident energies of the two probes, X-ray (for XPS) and electron (for EDS). The XPS provides the chemical composition at the surface region, whereas EDS effectively denotes the concentration of elements present at deeper layers, i.e., near-bulk property [40]. Accordingly, the obtained results confirmed the carbon incorporation at different depths of the catalyst surface. The doped carbon is distributed on the surface of CTiO_2_ and BTiO_2,_ as well occupying interstitial positions in TiO_2_ lattice.

#### 2.1.5. FT-IR Analysis 

FTIR analysis was conducted in order to investigate the surface properties of the catalysts (Figures S2 and S3). As can be seen for both BTiO_2_ and CTiO_2_, the low FT-IR frequencies at 600–900 cm^−1^ are assigned to Ti-O-Ti bridge stretching modes and Ti-O bond [41,42]. The broad band at of 3000-3500 cm^−1^ matches the surface hydroxyl stretching [43]. The peak observed at 1635 cm^−1^ for BTiO_2_ and CTiO_2_ corresponds to the vibrations of OH bonds of surface-adsorbed water molecules on the surfaces of the catalysts [44]. Interestingly, new carbon bands at 1310 cm^−1^ and 1090 cm^−1^ were observed for BTiO_2_ and CTiO_2,_ respectively, due to C-O stretching, thus providing clear evidence for the successful incorporation of carbon into the structural lattice of the modified catalyst.

### 2.2. Antifouling Coatings Performance in Seawater

**Figure 5:**
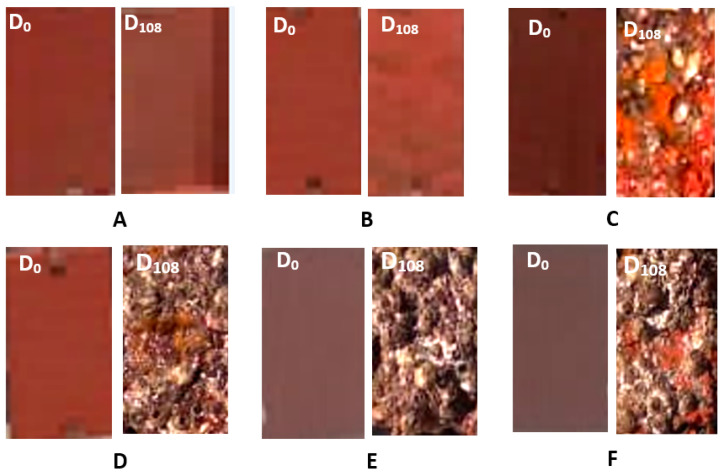
Photographic pictures of the coated panels with: (**A**) BTP; (**B**) CTP (**C**) LTiO_2_, (**D**) HTiO_2_, and (**E**) STiO_2_, and (**F**) commercial paint, from the beginning (Day 0) until the end of immersion period (Day 108).

The antifouling performance of the prepared coatings was evaluated during their exposure time in seawater for 108 days, starting from 20/5/2021. The photographic inspection of the coated panels was taken from the beginning (Day 0) until the end of the immersion period (Day 108). As can be clearly noted from Figure 5, no fouling organisms have been observed on the surface of the panels coated with CTP and BTP paints after 108 days of immersion in the Eastern Harbour seawater of Alexandria, Egypt. Both CTP and BTP paints demonstrated an excellent antifouling performance toward fouling organisms in the 108-day sea immersion period. The new paint formulations inhibited the settlement and controlled the adhesion of fouling organisms onto the immersed panels. In contrast, the surfaces of both blank (coated with commercial paint) and control (unpainted) steel panels showed heavy fouling, with barnacles attached to the surfaces of the panels.

Interestingly, these promising results were obtained during spring and summer seasons, which are considered the highest accumulation seasons for fouling organisms in the Eastern Harbour of Alexandria [45].

Nearly the same results were obtained from the marine algae *Grateloupia filicina* that showed the best results, followed by *Corallina mediterranea* after immersion in the EH for 140 days [46].

Basically, several marine creatures defend themselves against biofouling. Surface microstructures and surface wettability were suggested as potential mechanisms against biofouling [47,48,49]. Many secondary metabolites excreted by marine organisms have shown significant power to repel or deter biofouling organisms. In this context, several Rhodophyta species (red algae) were extracted and fractionated for the isolation of antifouling agents that are safe for the environment and do not kill fouling organisms. Regarding antifouling extracts, Rhodophyta are the second best investigated macroalgae. Antifouling metabolites isolated from the red algae mainly belong to terpenoids, especially halogenated and nonhalogenated sesquiterpenes, diterpenes, and phenols. Polar extracts (either EtOH or MeOH) displayed better antifouling activity than their corresponding non-polar extracts [49]. These antifouling extracts displayed potent activity against diatoms, microbes, and algal spore settlement. A survey of the literature revealed that the genus *Bostrychia* is a rich source of several polar substances such as amino acids, brominated sulphated phenols, and terpenoids [50,51,52,53,54,55,56,57]. As might be concluded, the antifouling activity of the polar extract of *B. tenella* could be partly attributed to the presence of polar function groups including amino, hydroxyl, carbonyl, carboxyl, carbon-carbon double bond, carbon-halogen, and hydrogen-bonding functionalities (Figure S4) [58].

**Figure 6:**
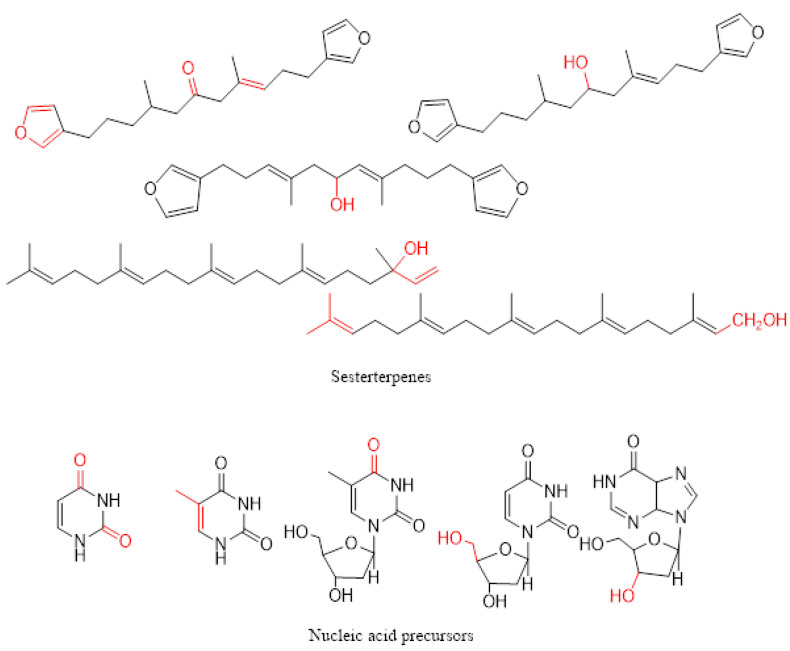
Secondary metabolites isolated from *Carteriospongia foliascens* (some selected active functionalities are coloured red).

**Figure 7:**
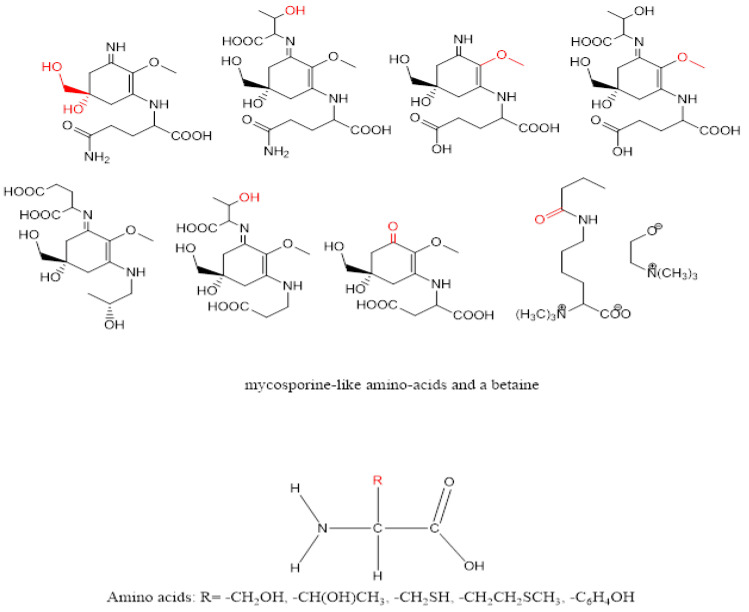
Secondary metabolites isolated from *Bostrychia tenella* (some selected active functionalities are coloured red).

The second marine organism examined in this work is the marine sponge *C. foliascens*. Despite the relatively large surface area of this whorled or curved plate-like or cup-like marine creature, it does not suffer from bioaccumulation of the fouling-producing organism on its surface. This antifouling behaviour is attributed to its ability to produce secondary compounds with deterring or repelling power against the fouling organisms. Sponges of the genus *Carteriospongia* were phytochemically examined for its chemical constituents by many research groups and were found to be a warehouse of sesterterpenoids, fatty acid derivatives, and nucleic acid precursors [59,60,61,62,63,64]. One of the coauthors studied the secondary metabolite contents of the Red Sea sponge *Carteriospongia foliascens* and identified several terpenoids and steroids including isofurospongin-2 (Figures S5 and S6), furospongin-1 (Figures S7 and S8), and 5α,8α—epidioxyergost-6-en-3β-ol (Figures S9 and S10) [65]. Figure 6 and Figure 7 illustrate the presence of several polar functionalities, such as N-H, O-H, and C-H, H-bonding in *C. foliascens* (Figure S11) and *B. tenella*, respectively. Several scientific reports showed that these polar groups enhanced the antifouling activity of the organic extract [49].

Therefore, based on the previously mentioned considerations, the incorporation of carbon into the lattice of both BTP and CTP during the synthesis process has been successfully achieved via the addition of natural organic residues of marine extracts of *Bostrychia tenella* and *Carteriospongia foliascens*, respectively, which has been evidenced by EDS and XPS analysis. This novel combination resulted in excellent antifouling properties in an ecologically relevant way.

### 2.3. Physicochemical Parameters of Seawater

It is critical to investigate the influence of the fabricated paint formulations on the physicochemical parameters of the seawater surrounding the immersed coated panels. The physicochemical parameters of the EH seawater are presented in Table 2. The EH’s seawater temperature ranged from 24.7 to 30.2 °C with a mean of 27.26 ± 1.87 °C. The pH ranged between 8.01 and 8.41 showing that it is normal as it lays in the alkaline side. Salinity fluctuated between 37.30 and 37.80 PSU/ppt with a mean of 37.65 ± 0.21 PSU/ppt. Transparency was mostly clear as the depth of the Eastern Harbour is 150 cm. The minimum transparency value was 144 cm while the maximum value was 149 cm, with a mean value of 147.5 ± 1.87 cm. With respect to the alkalinity, it ranged between 3.25 and 3.45 meq/L, with a mean of 3.37 ± 0.07 meq/L. Dissolved oxygen (DO) ranged between 3.25 and 4.13 mLO_2_/L with a mean of 3.74 ± 0.31 mLO_2_/L indicating low DO concentrations during the studied period, which may be due to the eutrophic conditions of Eastern Harbour seawater [45], beside the observed higher temperature during the summer season. Oxidizable organic matter (OOM) concentrations fluctuated between 1.913 and 2.857 mgO_2_/L with a mean of 2.481 ± 0.357 mgO_2_/L. Nitrite (NO_2_^−^) concentrations varied between 0.075 and 1.450 µM with a mean of 0.996 ± 0.486 µM. Nitrates (NO_3_^−^) differed between 13.94 and 15.32 µM with a mean of 14.52 ± 0.455 µM. Ammonium (NH_4_^+1^) concentrations fluctuated between 3.15 and 4.65 µM with a mean of 3.95 ± 0.49 µM. Phosphate (PO_4_^3−^) concentrations varied between 2.15 and 3.75 µM with a mean of 3.12 ± 0.55 µM. Silicate (SiO_3_^−^) concentrations fluctuated between 4.75 and 5.65 µM with a mean of 5.22 ± 0.32 µM. Finally, sulphate (SO_4_^2−^) concentrations varied between 3.495 and 3.656 g/L with a mean of 3.541 ± 0.075 g/L.

As can be seen from the studied physicochemical parameters of the EH seawater, the immersed coated panels have no discernible effect on the various seawater characteristics. Despite the eutrophic conditions of the surrounding seawater, which accelerate the fouling process [66], the promising antifouling performance of the new paint formulations (BTP and CTP) is clearly evident.

### 2.4. Mechanism for Marine Extract Mediated Synthesis of TiO_2_ Nanoparticles

The secondary metabolites that possess anionic radicals, such as terpenoids, polyphenols, sugars, alkaloids, phenolic acids, and proteins, play an important role in the bio-reduction of the metal ions, yielding metallic nanoparticles [67,68]. Examples of the main types of the secondary metabolites of *C. foliascens* and *B. tenella* proposed to reduce the metal ions are shown in Figure 6 and Figure 7, respectively.

**Figure 8:**
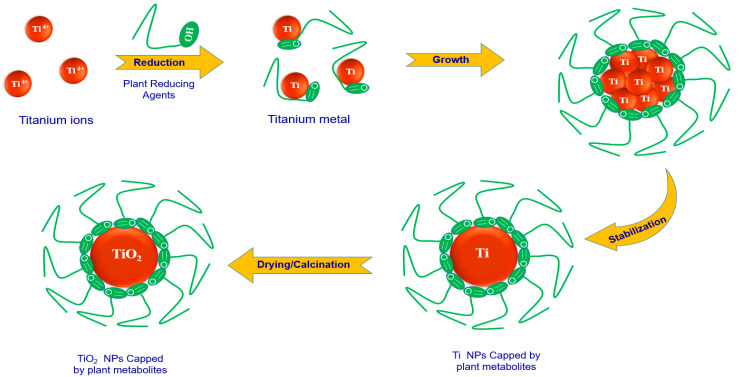
Schematic diagram for mechanism of the synthesis of TiO_2_ nanoparticles by a marine extract.

Generally, the biosynthesis of plant-based metallic nanoparticles can be suggested to be accomplished in three consecutive phases: reduction phase, growth phase, and stabilization phase [69]. The mechanism of marine extract (alga/sponge) mediated synthesis of TiO_2_ nanoparticles can be described by considering these phases as follows (Figure 8):

1. Reduction phase: involves the reduction of Ti^4+^ ions and the reduced Ti atoms undergo nucleation. This phase is the most important one, wherein the Ti ions (Ti^4+^) are recovered from their salt precursor (Titanium (IV) butoxide) through the interaction of marine extract (alga/sponge) secondary metabolites. These metabolites including alkaloids, flavonoids, polyphenols, and terpenoids can act as chelator to Ti^4+^. Mostly the hydroxyl function (OH^−^) of the metabolites content develops coordination with metal ions and donates an electron for the reduction process. The Ti metal ions are transferred from +4 oxidation state to zero-valent state, and then nucleation of the reduced Ti atoms occurs;
2. Growth phase: involves the spontaneous coalescence of small adjacent Ti nanoparticles into larger size nanoparticles, that is, Ostwald ripening (a process in which nanoparticles are directly formed through heterogeneous nucleation and growth and further reduction of metal ion). The aggregation occurs because of stronger binding energy between Ti metal atoms as compared to atom-solvent binding energy. This process enhances the thermodynamic stability of Ti nanoparticles;
3. Stabilization phase: is the final phase in the biosynthesis of TiO_2_ nanoparticles. Nanoparticles acquire the most energetically favorable conformation, with this process being strongly influenced by the ability of the alga/sponge extracts to stabilize metal nanoparticles. Ti nanoparticles eventually get their most intensely favorable and steady morphology when capped through marine alga/sponge metabolites, which prevents further aggregation of metal nanoparticles. After drying and calcination processes, the final TiO_2_ nanoparticles, capped with the bioactive metabolites, were obtained. The presence of these biologically active capping metabolites has been confirmed by the detection of the surface functional groups such as C-C, C-O/C=O and O-C=O, derived from these compounds of the marine alga/sponge extracts, as illustrated by XPS analysis of both BTiO_2_ and CTiO_2_. These capping bioactive compounds are presumed to increase the stability of the nanoparticles as well as enhance their antifouling efficiency.

### 2.5. Antifouling Mechanism

**Figure 9:**
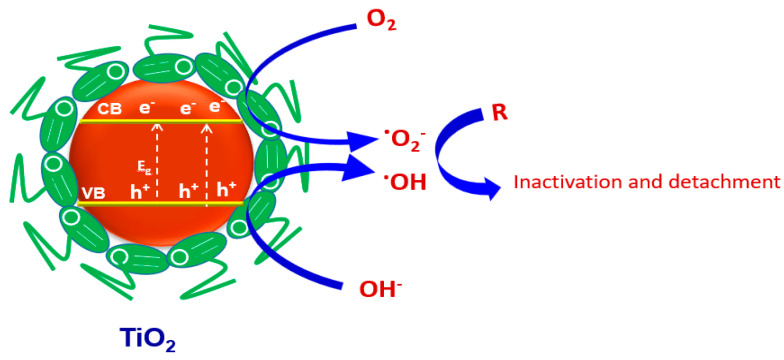
Schematic diagram for antifouling mechanism.

Figure 9 illustrates the simplified antifouling mechanism using the as-synthesized catalysts. When catalyst (BTiO_2_ or CTiO_2_) is irradiated with light of energy (hν) ≥ its band gap energy (E_g_ = 2.9 eV), hole-electron pairs are produced (Equation (2)), as well as the remaining holes ($h_{\begin{array}{c} vb \end{array}}^{+}$) in the valence band and the electrons ($e_{cb\;}^{-}$) in the conduction band. Consequently, two types of reactions can occur: electron-mediated reduction and oxidation reaction by holes. The positively charged holes ($h_{\begin{array}{c} vb \end{array}}^{+}$) capture the surface OH^−^ groups as well as interact with water molecules to generate the reactive hydroxyl radicals (^•^OH). Meanwhile, the dissolved oxygen molecules are reduced by the photogenerated electrons ($e_{cb\;}^{-}$) to form superoxide radicals $\;({\dot{O}}_{\begin{array}{c} 2 \end{array}}^{-})$, which can either directly degrade the attached organic molecules (R) adsorbed onto the photocatalyst surface which cause fouling (Equation (8)), or via multistep reactions, and reactions with H_2_O to further initiate the generation of strong oxidizing hydroxyl radicals (^•^OH) (Equations (5)–(7)). The generated reactive ^•^OH radicals react with organic molecules ((Equation (8)) [70,71], leading to inactivation of fouling activities.

(2) $${TiO}_{2}+\;hv\;\to \;e_{cb\;}^{-}+h_{\begin{array}{c} vb \end{array}}^{+}$$

(3) $${\;h}_{\begin{array}{c} vb \end{array}}^{+}+OH^{-}\to \dot{O}H$$

(4) $${\;h}_{\begin{array}{c} vb \end{array}}^{+}+H_{2}O\;\to \dot{O}H+H^{+}$$

(5) $$\;{\dot{O}}_{\begin{array}{c} 2 \end{array}}^{-}+\;H^{+}\to \dot{OO}H$$

(6) $$\dot{OO}H+\;H^{+}+e_{cb\;}^{-}\;\to H_{2}O_{2}$$

(7) $$H_{2}O_{2}+e_{cb\;}^{-}\to \dot{O}H+OH^{-}$$

(8) $${\dot{O}}_{\begin{array}{c} 2 \end{array}}^{-},\;\;\dot{O}H+R\to Incativation\;of\;fouling\;activities$$

## 3. Materials and Methods

### 3.1. Extraction of the Marine Samples

#### 3.1.1. Extraction of the Red Sea Algal Materials

The algal material of *B. tenella, Laurencia obtusa* (Ceramiales, Rhodomelaceae), *H. tuna*, and *S. filipendula* were collected in Sep. 2020 from the Red Sea, off Rabigh coast, Saudi Arabia (21°29′31″ N; 39°11′24″ E). The samples were identified by Prof. Mohsen El-Sherbiny, faculty of Marine Sciences, KAU, Jeddah, Saudi Arabia. The reference standards of the four algae species (JAD 03110, JAD 03111, JAD 03112, and JAD 03110, respectively) were deposited at the Faculty of Marine Sciences, King Abdulaziz University (Jeddah, Saudi Arabia). Of each alga material, 100.0 g was air-dried, then extracted with methanol (1000 mL, three times). The residues (2.85, 2.45, 2.67, and 2.96 g, respectively) were filtered and kept in a deep freeze at −10 °C for 24 hrs. The extracts were filtered again to remove the fatty materials, the filtrates were then evaporated separately until dry, and yielded greenish residues (2.4, 2.13, 2.23, and 2.60 g, respectively).

#### 3.1.2. Extraction of the Red Sea Sponge Carteriospongia Foliascens

The fresh sponge material, *Carteriospongia foliascens* (Dictyoceratida: Thorectidae) was collected in Sep. 2020 from the Red Sea, Salman Gulf, Jeddah, Saudi Arabia (21°51′39.8″ N; 38°58′42.7″ E). The reference standard (JAD 04090) was deposited at the Faculty of Marine Sciences, King Abdulaziz University (Jeddah, Saudi Arabia). The sponge material (75.0 g) was extracted with methanol (500 mL, three times). The residue (12.1 g) was filtered and kept in a deep freeze at −10 °C for 24 h. The extract was filtered again to remove the fatty materials, the filtrate was then evaporated until dry, and yielded a greenish-brown residue (8.9 g).

### 3.2. Green Synthesis of TiO_2_ Nanoparticles

Titanium (IV) butoxide (TBT) was used as a precursor of Ti, 20 mL of TBT was slowly added into an equal volume of absolute ethanol. Of each of the marine extracts, 2.0 g was dissolved in 25 mL ultrapure Milli-Q water, and then added dropwise to the titanium solution at 50 °C under vigorous stirring for 2 h, then the formed solution was transformed into gel by aging for 24 h. The desired TiO_2_ nanoparticles were formed by drying the gel at 100 °C for 12 h, followed by grinding in a crystal mortar pestle to obtain the final powder form of TiO_2_ nanoparticles, and finally, calcination of the nanoparticles in a muffle furnace at 500 °C for 2 h. 

### 3.3. Characterization of BTiO_2_ and CTiO_2_

X-ray diffraction (XRD) analysis of the synthesized TiO_2_ was carried out by using a Rigaku Ultima IV X-Ray diffractometer with Cu*K*_α_ radiation at 40 kV and 40 mA. The diffractogram was recorded at a scan rate of 4.0° min^−1^ over the 2θ range of 20–80 degrees. To study the surface morphology and elemental composition of the photocatalysts, Scanning Electron Microscope (SEM, A JSM-7600F, JEOL, USA) with an Energy Dispersive X-Ray Spectroscopic unit (EDS, X-Max 50 mm^2^, Oxford Instruments). Surface composition of the photocatalysts was analyzed by X-ray photoelectron spectroscopy (XPS, SPECS) operating at a base pressure of 4 × 10^−10^ mbar, using Mg-Kα (1253.6 eV) X-ray source at 13.5 kV, 150 W of X-ray power. The adventitious carbon C1s at 285 eV corresponding to C-C bond has been used for all XPS spectra as a binding energy reference for charge correction. The spectrometer was calibrated to the position of the 3d5/2 line of sputter-cleaned silver sample with binding energy 368.26 eV. Perkin Elmer Fourier transform infrared spectrometer (FTIR) was used to confirm the functional groups, which are present in the photocatalysts. The scanning process was conducted in the range of 400–4000 cm^−1^_._

### 3.4. Fabrication of a New Marine Antifouling Paint Formulation

Novel marine paint formulations were prepared by mixing of 300 g oil binder material, 100 g iron oxide, 100 g zinc oxide, 100 g PVC, and 100 g complementary pigment in the presence of 300 g of xylene as a solvent by using ball mill until complete mixing was attained. Then, 0.1 g of each synthesized nanoparticles was added into 10 g of the prepared paint formulation under continuous mixing to form the desired antifouling coating formulation. Marine paint formulations derived from BTiO_2_, CTiO_2_, LTiO_2_, HTiO_2_, and STiO_2_ are denoted as BTP, CTP, LTP, HTP, and STP, respectively. 

### 3.5. Preparation of Steel Panels

**Figure 10:**
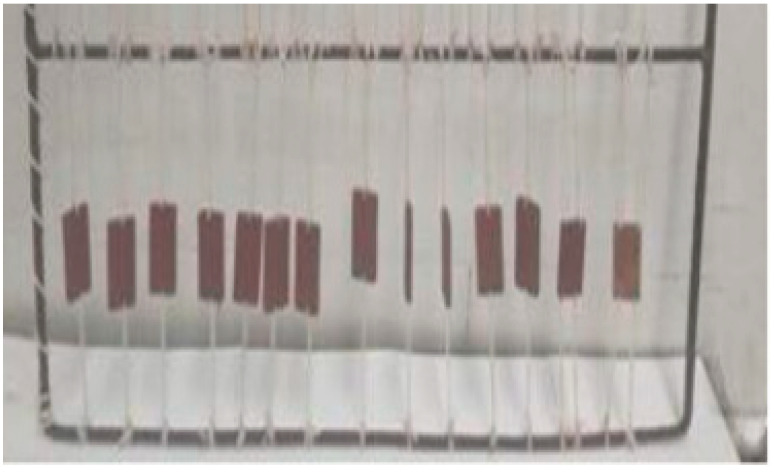
Coated steel panels with the prepared marine paint formulations before immersion.

A steel frame with a dimension of 100 cm × 80 cm was used to hang the steel panels using nylon thread (Figure 10).

### 3.6. Paints Applications

Two successive brush coatings of each of the prepared antifouling paints (BTP, CTP, LTP, HTP, and STP) were applied twice to the steel panels, allowing sufficient time after each coating to become dry. For comparison, a coated panel with a commercial marine paint formulation was utilized as a blank, whereas an uncoated panel was used as a control. The weights of the paint films on steel panels are shown in Table 3.

### 3.7. Panel’s Immersion Test

**Figure 11:**
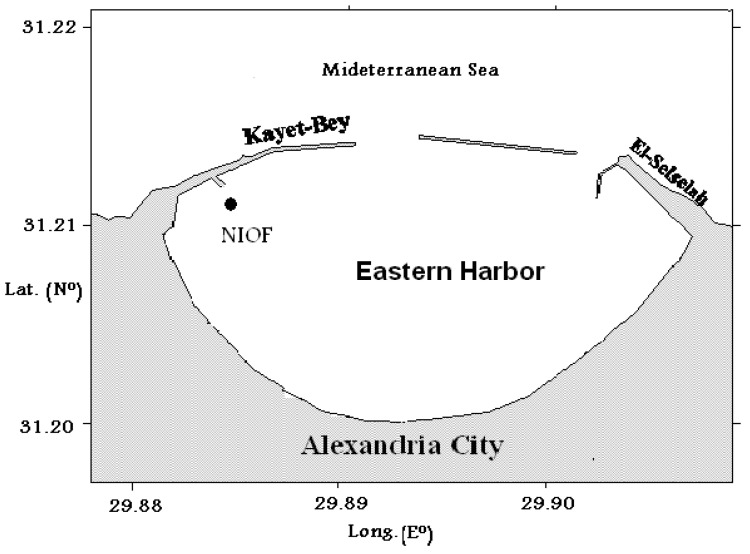
Location of panels’ immersion in the Eastern Harbour of Alexandria, Egypt.

The steel panel immersion test was carried out to study the efficacy of the prepared new formulations as antifoulants. The frame containing the coated panels was directly immersed in the Eastern Harbour (EH) seawater of Alexandria, Egypt on 20 May 2021. The map of the immersion location is shown in Figure 11. Following the immersion of the frame containing the coated panels in seawater, the surfaces of the panels were visually and photographically inspected for fouling succession at various time intervals. The performance of prepared coatings was evaluated during their exposure time in seawater for 108 days.

### 3.8. Physicochemical Parameters Measurements of Seawater

The physicochemical parameters of seawater starting on the immersion date and during following up of the panels were investigated. Water temperature was measured using an inductive portable thermometer. Salinity was measured using the Salinometer model Beckman RS-10-X3 range to about 0.1 units. Water transparency was measured using a Secchi disc.

After taking the necessary precautions in the sampling and standardization processes, the pH value of water samples was measured in situ to about 0.1 per unit using a portable pH-meter (Orion Research model 210 digital pH-meters). For dissolved oxygen (DO) determination, water samples were collected in 125 mL glass bottles and fixed with 1 mL manganous sulfate followed by 1mL iodide solution until the analysis in the laboratory. DO was determined according to the classical Winkler’s method, modified by FAO 1975 [72]. The amount of dissolved oxygen in each sample was calculated by applying the following equation [73]:

(9) $${mlO}_{2}L=\frac{N\times V\times 32,000/4}{4B\times 1.43}$$

*N* = Normality of sodium thiosulphate,
*V* = Volume of sodium thiosulphate
*B* = Volume of oxygen bottle

The alkalinity was determined according to standard methods [74]. The sample (10 mL) was titrated against 0.02 N HCl designated pH value (pH 4.5). Alkalinity was calculated from the following equation:

(10) $$\mathrm{Total}\;\mathrm{alkalinity}=\frac{\mathrm{mL}\;\mathrm{of}\;\mathrm{HCl}\;\times \;1000{\;\times \;N}_{\mathrm{HCl}\;}}{\;\mathrm{mL}\;\mathrm{of}\;\mathrm{sample}}$$

Oxidizable organic matter (OOM) was determined by permanganate oxidation method [64] and calculated from the following equation:

(11) $$\mathrm{mg}\;O_{2}/L\;(\mathrm{OOM})=\frac{\left(V_{\mathrm{blank}}-{\;V}_{\mathrm{sample}}\right)\times 8\times 1000\times N_{{\mathrm{Na}}_{2}S_{2}O_{3}}}{V\;\mathrm{of}\;\mathrm{sample}}$$

The most important nutrient salts which are the dissolved inorganic forms of nitrogen (NO_2_^−^, NO_3_^−^, and NH_4_^+^), phosphate (PO_4_^3−^), and silicate (SiO_3_^−^) were determined calorimetrically [75]. The absorbance was measured by using double-beam spectrophotometer model Shimadzu UV-150-02 and the values were expressed as µM. Sulphate (SO_4_^2−^) was precipitated as barium sulphate and measured turbidimetrically [76].

## 4. Conclusions

This study demonstrated that several Red Sea marine organisms, including red, brown, and green algae, along with a common soft-bodied Dictyoceratida sponge, *C. foliascens*, provide potent antifouling activity. 

Moreover, these marine organisms can be utilized as a scaffold for the green synthesis of nanoparticles. Regarding marine algae, the literature survey revealed that red algae are the most prolific source of secondary metabolites with potent antifouling activities. Brown algae came in the second stage, followed by green algae. 

These data from the literature are consistent with our finding that the currently examined red algae, *B. tenella* and *L. obtusa*, exhibited the most noticeable antifouling activities among all examined algae. The Red Sea sponge Carteriospongia foliascens lacks calcareous or silaceous spicules, which can deliver structural rigidity and provide defense against marine predators. The defense mechanism of these sponges evidently relies on a chemical defense strategy that embraces a battery of sesterterpenes. In this work, novel antifouling marine paint formulations have been successfully fabricated by employing titanium dioxide (TiO_2_) nanoparticles via a novel eco-friendly, green chemistry approach using the marine natural extracts of two red algae, *B. tenella* (Ceramiales, Rhodomelaceae), and *Laurencia obtusa*, a green alga, *H. tuna*, a brown alga, *S. filipendula*, and the marine sponge *C. foliascens*. The prepared paints using the extract of *B. tenella* and *C. foliascens* exhibited an excellent antifouling performance toward fouling organisms after 108 days of immersion of the coated steel panels in seawater. Interestingly, no effects were observable of the immersed coated panels on the physicochemical parameters of the surrounding seawater, thus revealing the safeness of the new paint formulations. By contrast to many synthetic antifouling additives and paints, which showed toxic effects to the microorganisms and barnacles, the prepared nanoparticles showed neither measurable effects on the water body surrounding the coated steel panels, nor killing behaviour towards marine animals that tried to attach themselves to the painted steel. The authors believe that the outputs of this study can be utilized as an environmentally secure, bioassay-guided strategy to isolate pure, potentially active metabolites, and as a starting point for integrating nano-sized advantages with benign source materials.

## Figures and Tables

**Table 1:** Optical properties of CTiO_2_ and BTiO_2_ nanoparticles.

Catalyst	Crystal Phase	Crystalline Size (nm)	EDS (Atomic%)			XPS (Atomic%)		
			Ti	O	C	Ti	O	C
**CTiO**_**2**_	Anatase	8.3	29.82	53.27	16.91	22.9	53.4	23.7
**BTiO**_**2**_	Anatase	22.86	33.50	52.90	13.60	19.5	66.3	14.2

**Table 2:** Physicochemical parameters of the Eastern Harbour seawater from the beginning (Day 0) until the end of the immersion period (Day 108).

Days		Temp. (°C)	pH	Salinity (PSU/ppt)	Transparency (cm)	Alkalinity (meq/L)	DO (mL O_2_/L)	OOM (mg O_2_/L)	NO_2_^−^	NO_3_^−^	NH_3_	PO_4_^3−^	SiO_3_^−^	SO_4_^2−^ (g/L)
									(µM)					
0		24.7	8.21	37.3	148	3.45	3.60	1.913	1.250	14.39	3.15	3.75	5.65	3.587
20		26.3	8.19	37.5	149	3.42	3.88	2.230	0.940	13.94	3.89	3.21	5.31	3.489
40		26.7	8.01	37.8	147	3.25	3.91	2.473	1.240	14.56	4.01	3.52	5.42	3.459
60		27.4	8.13	37.7	148	3.35	4.13	2.653	1.450	14.32	3.96	3.15	5.24	3.656
80		28.3	8.41	37.8	144	3.35	3.64	2.875	1.025	14.57	4.21	2.94	4.96	3.565
108		30.2	8.32	37.8	149	3.42	3.25	2.743	0.075	15.32	4.65	2.15	4.75	3.487
	Max	30.2	8.41	37.8	149	3.45	4.13	2.857	1.450	15.32	4.65	3.75	5.65	3.656
	Min	24.7	8.01	37.3	144	3.25	3.25	1.913	0.075	13.94	3.15	2.15	4.75	3.495
	Average	27.26		37.65	147.5	3.37	3.74	2.481	0.996	14.52	3.98	3.12	5.22	3.541
	St dev.	1.87		0.21	1.87	0.07	0.31	0.357	0.486	0.455	0.49	0.55	0.32	0.075

**Table 3:** Weight of the paint film on wood panels coated with BTP, CTP, commercial paint (blank), and unpainted panel (control).

Panel	Weight before Painting (g)	Weight after Painting (g)	Weight of Paint Film (g)	Weight of Paint Film Per Unit Area (g/cm^2^)
**BTP**	8.705	11.880	3.175	0.063
**CTP**	7.861	11.055	3.194	0.064
**Blank**	8.658	10.474	1.816	0.036
**Control**	8.771	-	-	-

## Data Availability

The original data are available from the correspondent author on request.

## Supplementary Materials

The following supporting information can be downloaded at: https://www.mdpi.com/article/10.3390/md21020062/s1, Figure S1: Images of marine organisms: A- *Bostrychia tenella, B- Carteriospongia foliascens, C- Laurencia obtusa, D- Sargassum filipendula, and E- Halimeda tuna;* Figure S2: FTIR spectrum of CTiO_2_; Figure S3: FTIR spectrum of BTiO_2_; Figure S4: FTIR spectrum of *B. tenella* extract; Figure S5: ^13^C-NMR of isofurospongin-2 isolated from *C. foliascens;* Figure S6: ^1^H-NMR of isofurospongin-2 isolated from *C. foliascens;* Figure S7: ^13^C-NMR of furospongin-1 isolated from *C. foliascens;* Figure S8: ^1^H-NMR of furospongin-1 isolated from *C. foliascens;* Figure S9: ^13^C-NMR of 5α,8α—epidioxyergost-6-en-3β-ol isolated from *C. foliascens;* Figure S10: ^1^H-NMR of 5α,8α—epidioxyergost-6-en-3β-ol isolated from *C. foliascens;* Figure S11: FTIR spectrum of *C. foliascens* extract.
