# Supplementary material for: Green Synthesis of TiO2 Nanoparticles Using Natural Marine Extracts for Antifouling Activity
Source: Mar Drugs. 2023 Jan 19;21(2):62. doi: 10.3390/md21020062 (PMC9962051; doi:10.3390/md21020062)
Supplement: Supplementary file 1 [file marinedrugs-21-00062-s001.zip › marinedrugs-2136950-supplementary/marinedrugs-2136950-supplementary.pdf]

SUPPLEMENTARY MATERIAL

Green synthesis of TiO_2 nanoparticles using natural marine extracts for antifouling activity

Walied M. Alarif [1], Yasser A. Shaban [1,*], Mohamed I. Orif [1], Mohamed A. Ghandourah [1], Adnan J. Turki [1], Hajer S. Alorfi [2] and Hermine R. Z. Tadros [3]

[1] Department of Marine Chemistry, Faculty of Marine Sciences, King Abdulaziz University, Jeddah 21589, Saudi Arabia; welaref@kau.edu.sa (W.M.A.), ydomah@kau.edu.sa (Y.A.S.), mioraif@kau.edu.sa (M.I.O.), mghandourah@kau.edu.sa (M.A.G.), ajturki@yahoo.com (A.J.T.)

[2] Department of Chemistry, Faculty of Science, King Abdulaziz University, Jeddah 21589, Saudi Arabia; halorfi@kau.edu.sa

[3] National Institute of Oceanography and Fisheries, Kayet Bay, Alexandria, Egypt; herminetadros@gmail.com

Correspondence: ydomah@kau.edu.sa (Y.A.S.); Tel.: +966-595-670522

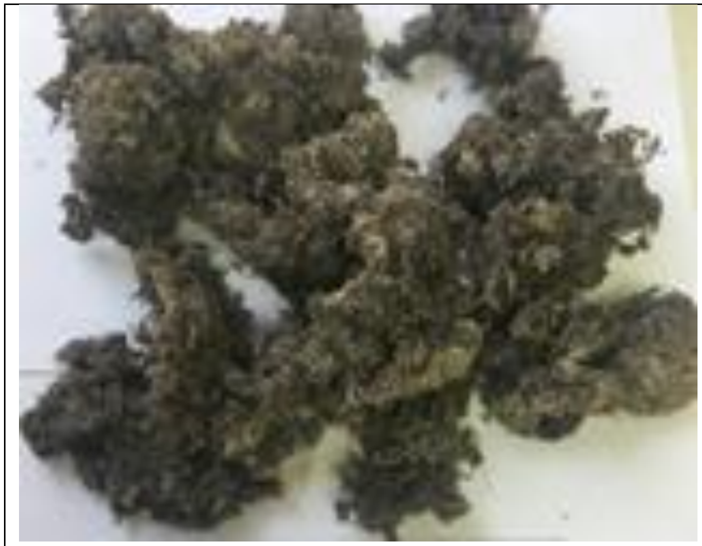

A- *Bostrychia tenella*

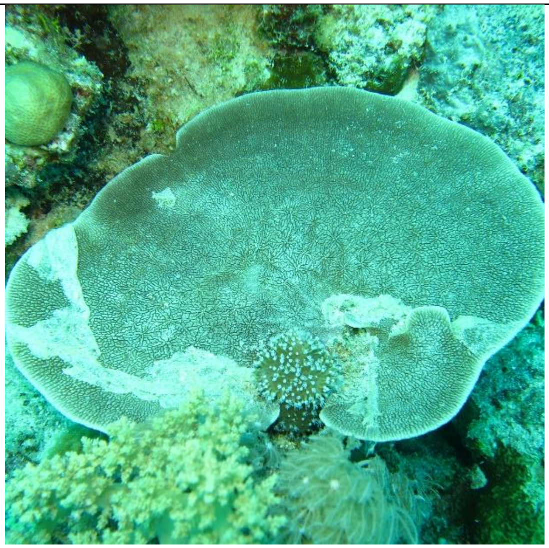

B- *Carteriospongia foliascens*

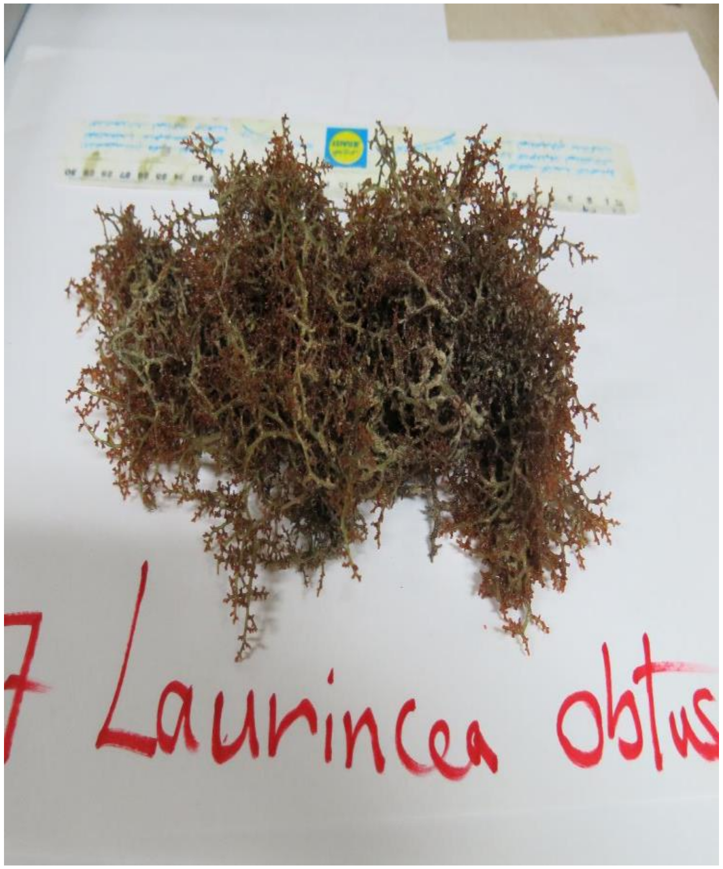

C- *Laurencia obtusa*

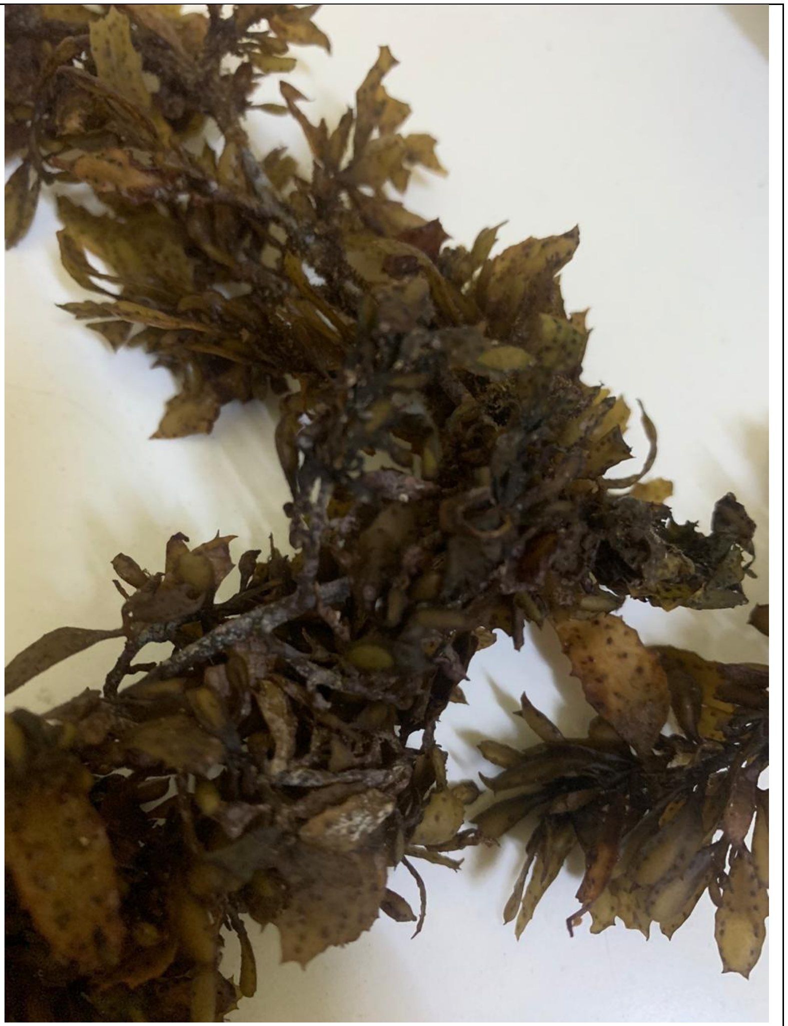

D- *Sargassum filipendula*

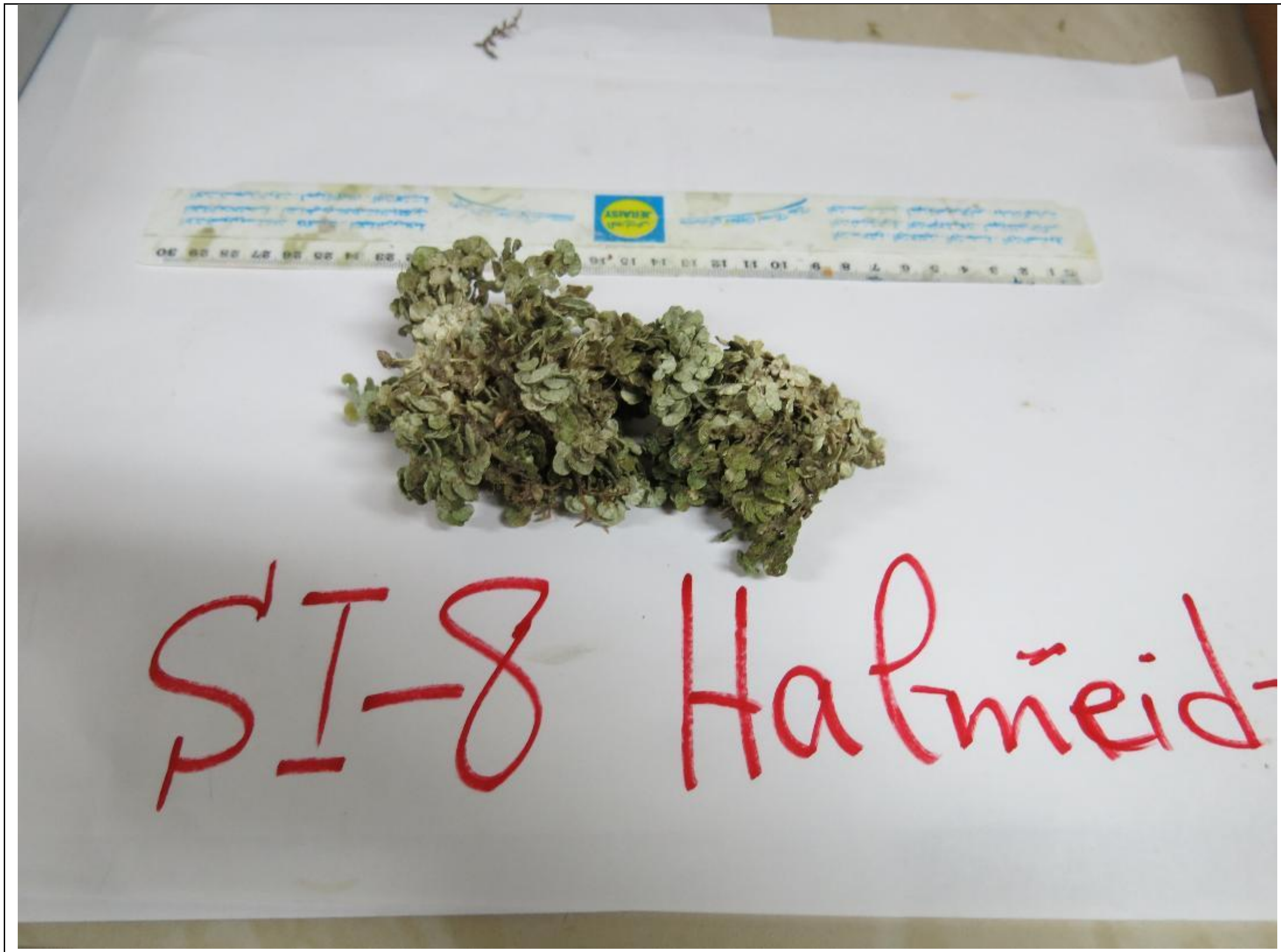

D- Halimeda tuna

Figure S1. Images of marine organisms: A- *Bostrychia tenella, B- Carteriospongia foliascens, C- Laurencia obtusa, D- Sargassum filipendula, and E- Halimeda tuna.*

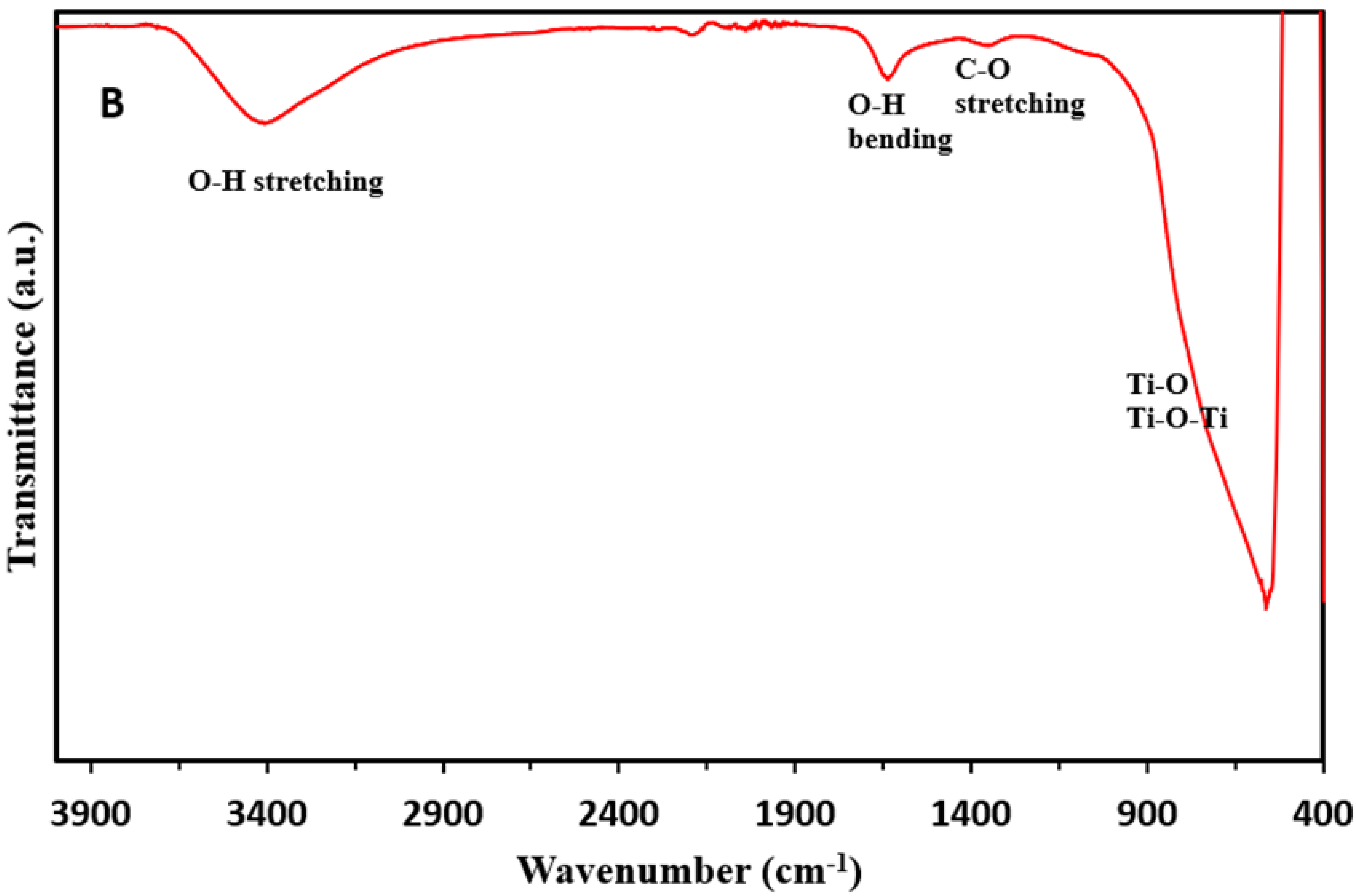

Figure S2. FTIR spectrum of $CTiO_2$.

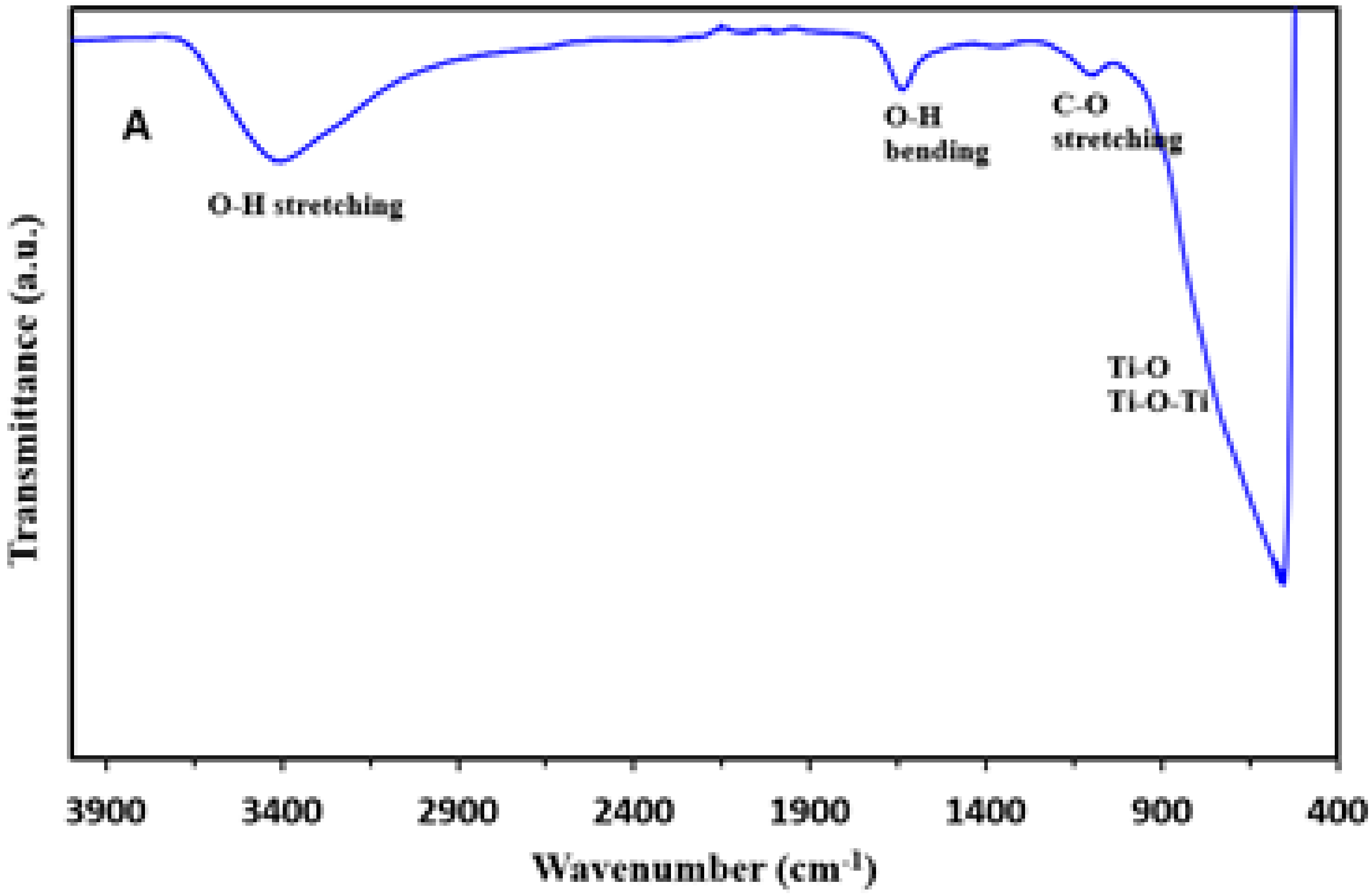

Figure S3. FTIR spectrum of $BTiO_2$.

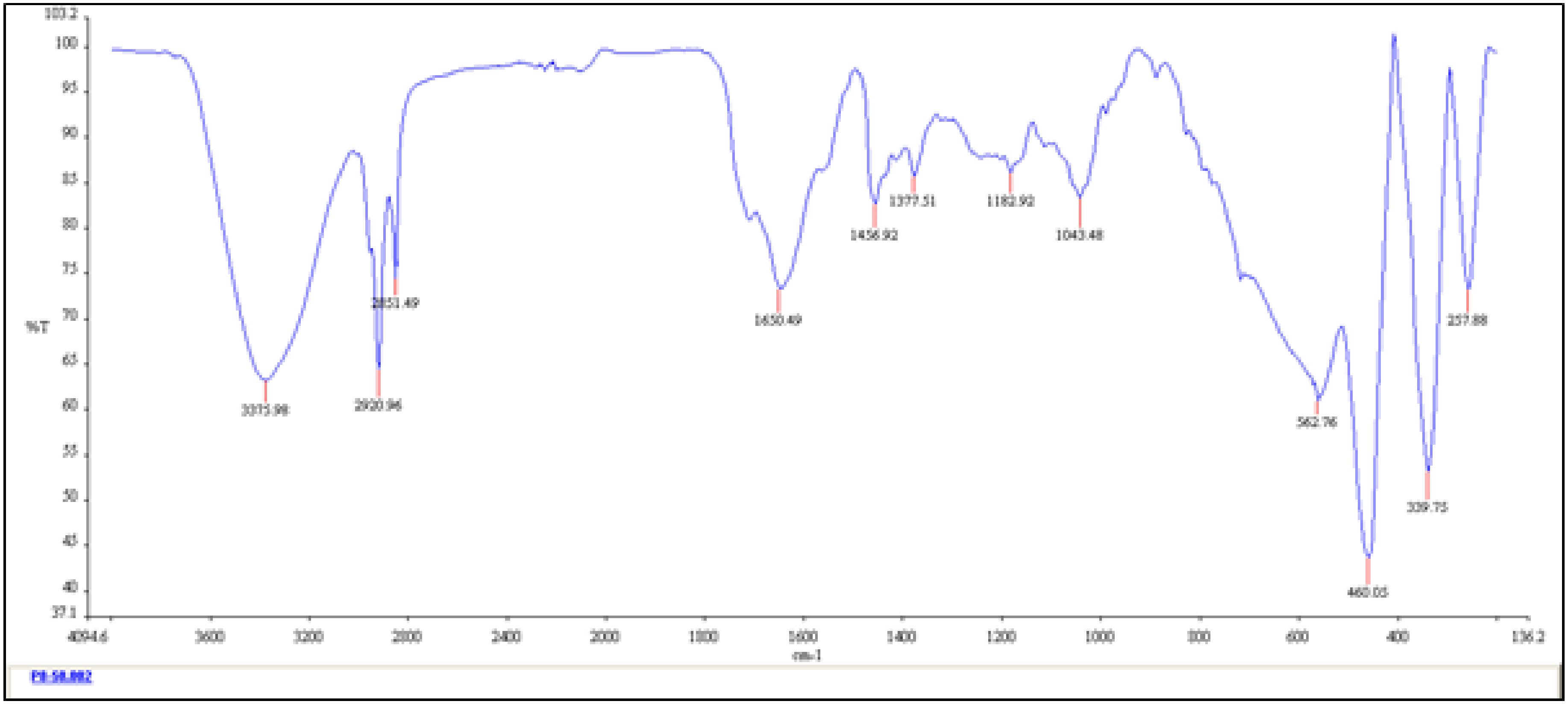

Figure S4. FTIR spectrum of *B. tenella* extract.

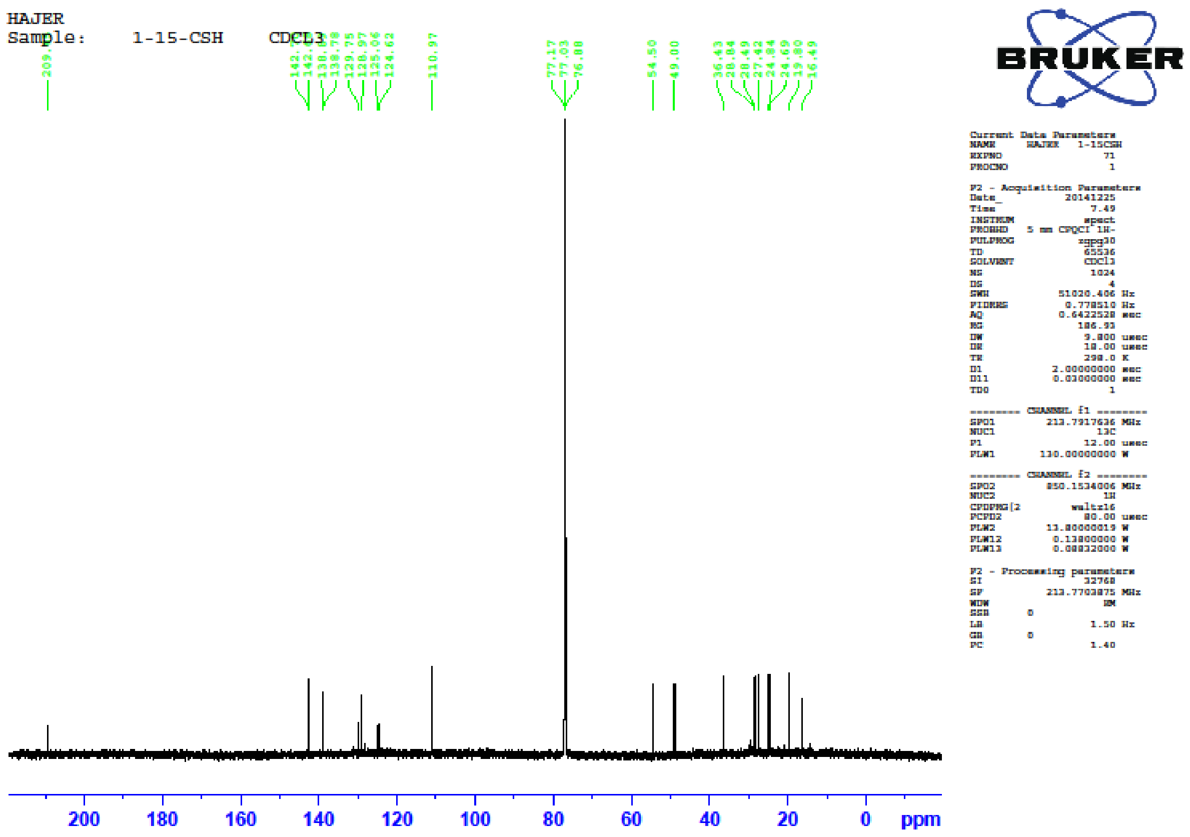

Figure S5. ^{13}C-NMR of isofurospongin-2 isolated from *C. foliascens*.

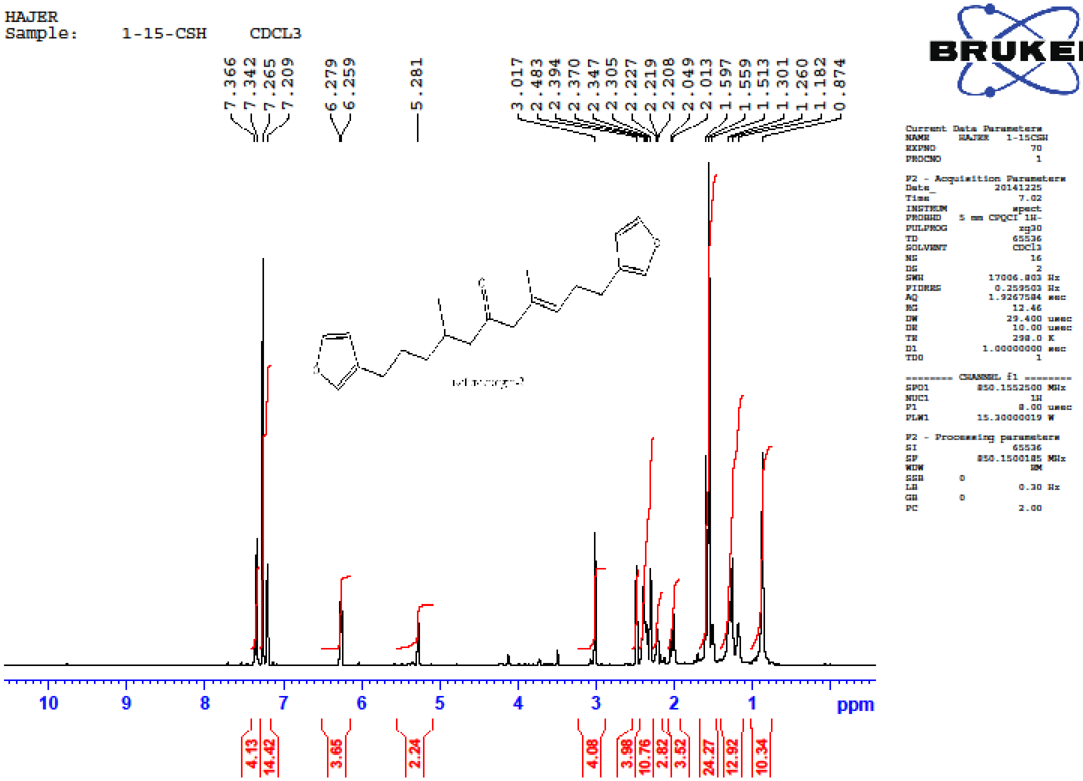

Figure S6. ^{1}H-NMR of isofurospongin-2 isolated from *C. foliascens*.

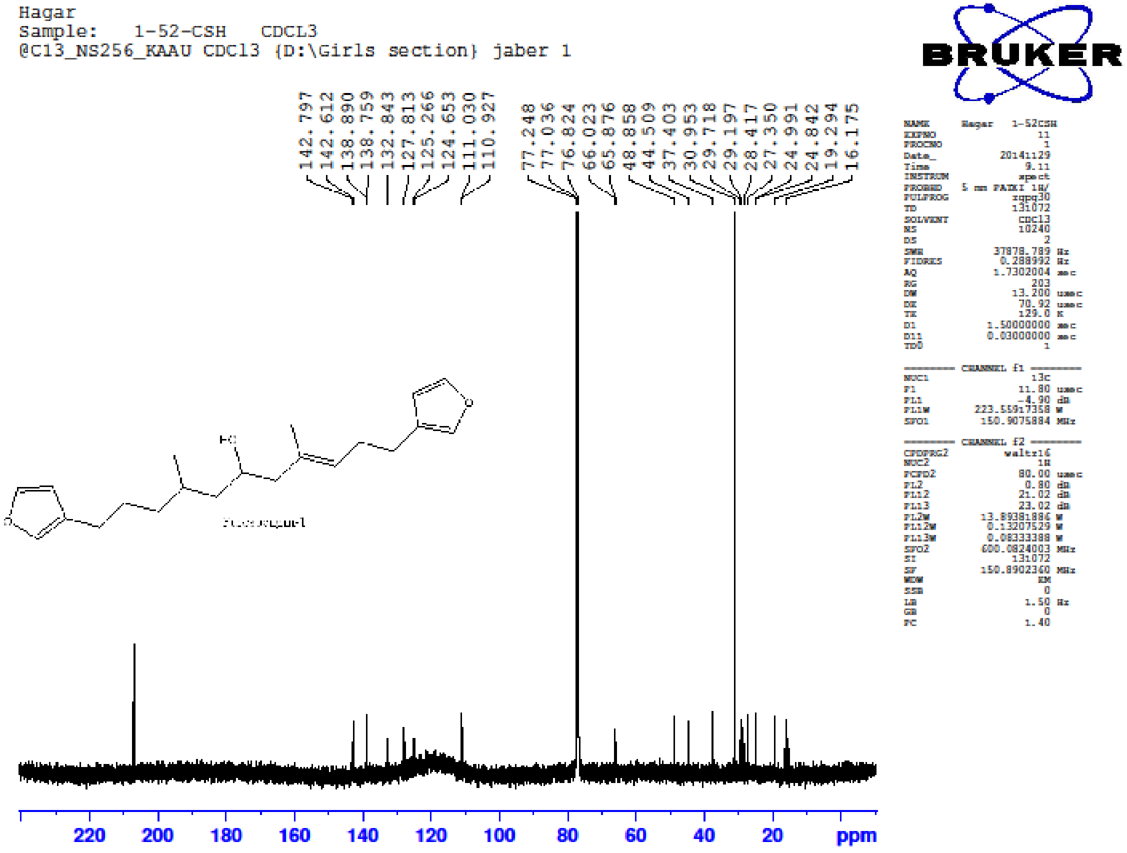

Figure S7. ^{13}C-NMR of furospongin-1 isolated from *C. foliascens*.

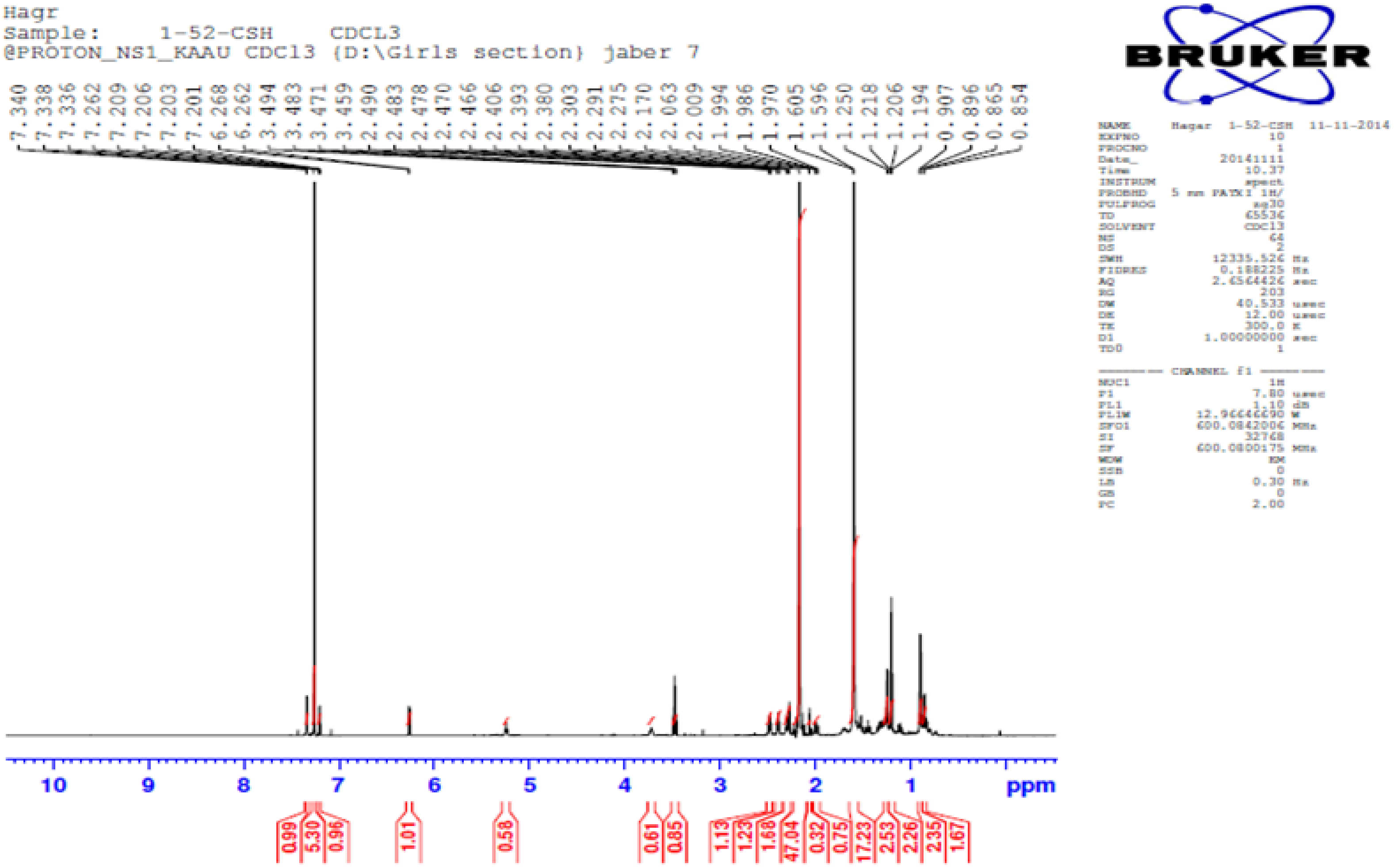

Figure S8. ^{1}H-NMR of furospongin-1 isolated from *C. foliascens*.

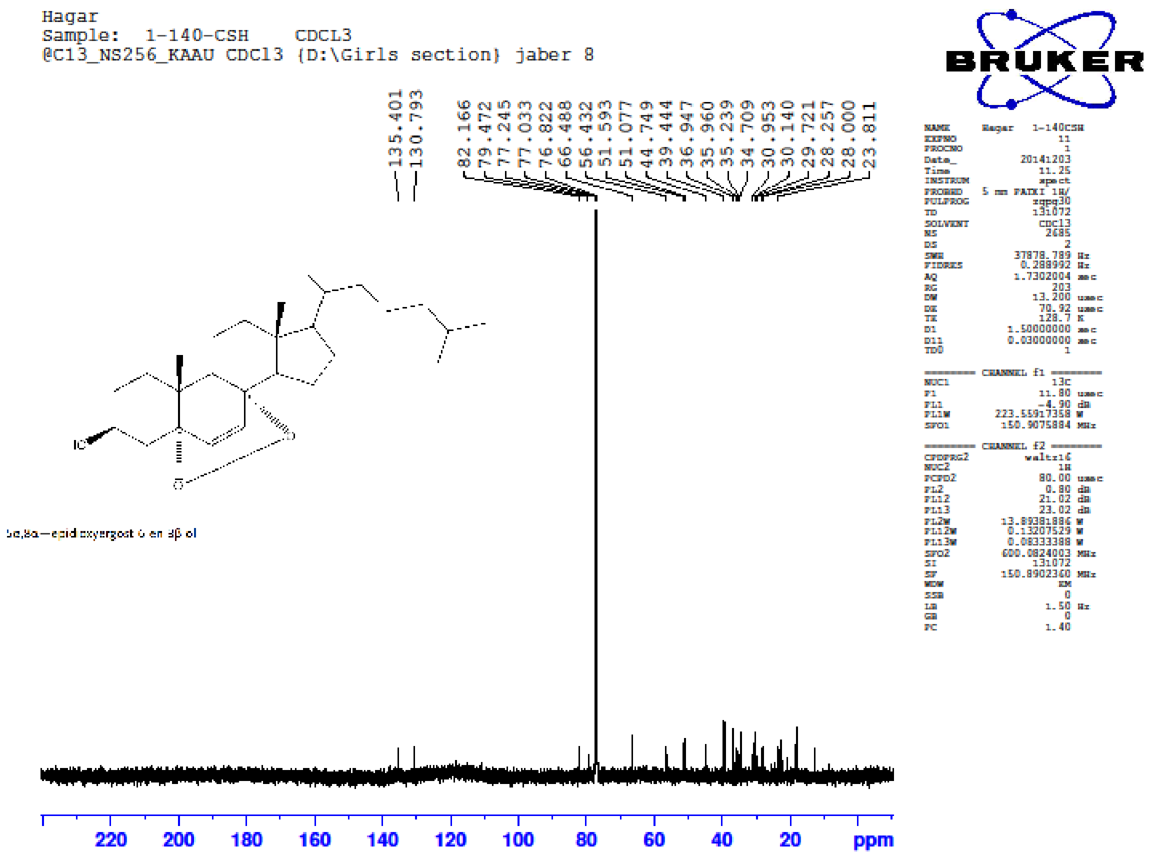

Figure S9. ^{13}C-NMR of 5α,8α—epidioxyergost-6-en-3β-ol isolated from *C. foliascens*.

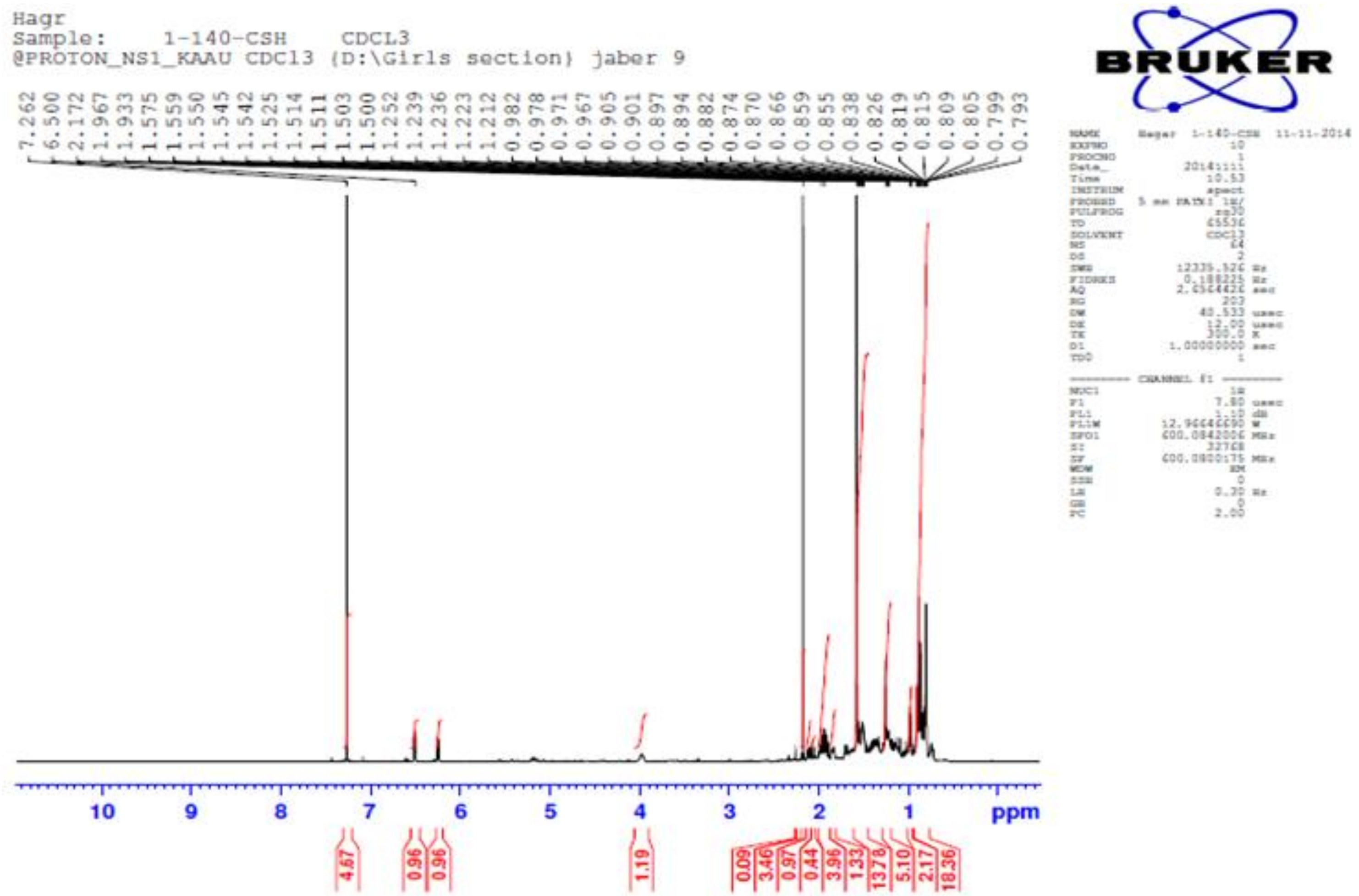

Figure S10. ^{1}H-NMR of 5α,8α—epidioxyergost-6-en-3β-ol isolated from *C. foliascens*.

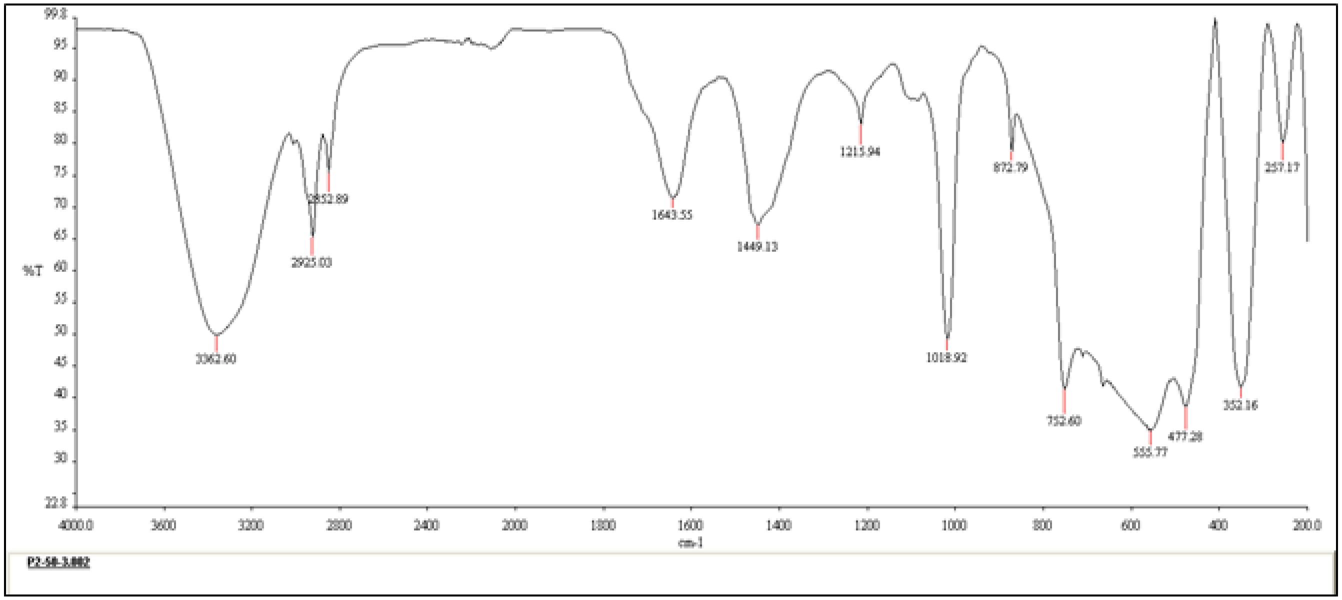

Figure S11. FTIR spectrum of *C. foliascens* extract.
